# What's Needed for a Migraine Cost‐Of‐Illness Study in Aotearoa New Zealand: Review of Data Sources and Gaps

**DOI:** 10.1002/snz2.70055

**Published:** 2026-05-18

**Authors:** Fiona Imlach, Sofiia Tsaregorodtseva, Maite Irurzun‐Lopez, William Leung, Calvin Chan, Ray Bose

**Affiliations:** ^1^ Department of Public Health University of Otago Wellington Wellington New Zealand; ^2^ Faculty of Medical and Health Sciences University of Auckland Auckland New Zealand; ^3^ University of Victoria Wellington New Zealand; ^4^ Department of Neurology Palmerston North Hospital Te Whatu Ora/Health NZ (MidCentral) Palmerston North New Zealand; ^5^ NeuralConnexion Specialist Neurology Services Auckland New Zealand

**Keywords:** cost of illness, data sources, migraine disease, New Zealand, prevalence, review

## Abstract

Cost of illness (COI) studies are used to quantify the economic burden of disease on health systems, employers and society, and identify where costs may be saved by improvements in disease management. Many migraine COI studies have been published internationally but none have included data from Aotearoa New Zealand (NZ), despite the known high prevalence and individual health burden of migraine. Adapting methods for data reviews developed at the University of Auckland, we reviewed known data sources to identify relevant NZ datasets containing information on migraine prevalence, direct and indirect costs from migraine, supplemented by searches of peer‐reviewed literature. Data sources were evaluated against pre‐defined criteria to assess quality. We found adequate data on migraine prevalence but limited to no data on migraine‐specific indirect costs (absenteeism or presenteeism, reduced employment or inability to work because of migraine). Data on direct costs were insufficient, due to poor or lack of diagnostic data in key areas such as primary care, pharmaceuticals, outpatients, laboratory or imaging tests. This review revealed significant data gaps that would need to be filled for a comprehensive and robust NZ migraine COI study or any ongoing research into migraine in NZ.

## 1 Introduction

Migraine in Aotearoa New Zealand (NZ) is estimated to affect 733,000 people (Global Burden of Disease Collaborative Network 2025a), although this is likely to be an undercount, given that around half of adults with recent migraine symptoms in NZ do not have a migraine diagnosis (Ministry of Health 2026). Globally, migraine is the second highest cause of disability from neurological conditions amongst adults under 60 years and the highest for children and adolescents (Peres et al. 2024), with health burden twice as great in females compared to males (Husøy and Steiner 2025). Despite being a non‐fatal condition, migraine ranks highly due to its prevalence, particularly among younger people, and the severity of symptoms during attacks, which can be functionally debilitating and extend beyond the headache phase. Migraine impacts on people's ability to work as well as having negative effects on mental health, physical health, family and social life (Garrett and Imlach 2024).

### 1.1 Issues with Migraine Care in NZ

Many studies have found that migraine is underdiagnosed and undertreated (Buse et al. 2021, Adams et al. 2023, Raggi et al. 2024), although effective treatments exist. In NZ, the availability of migraine‐specific medications (e.g. triptans for acute treatment of attacks and calcitonin gene–related peptide (CGRP) blockers for both acute and preventive treatment) is limited. As of March 2026, only two of the seven triptans used worldwide were available and funded in NZ (longer acting and nasal spray formulations not available in NZ); only four of the eight CGRP blockers used worldwide were available but none were funded. Similarly, neurostimulation devices, another recent treatment innovation (Yuan et al. 2025), are not available easily or at all in NZ, and onabotulinumtoxinA, a safe and effective treatment for chronic migraine, is difficult to access through the public health system. And although the recommended model of migraine care is for most patients with migraine to be managed in primary care, with escalation to secondary and tertiary (headache centre) care for chronic and complex cases (Steiner et al. 2021), this level of service is not consistent throughout NZ. There are few headache specialists and no formal headache centres, and access to neurologists is limited due to workforce shortages, with long waiting times and variation in the acceptance of headache referrals to public outpatient clinics throughout the country (Imlach 2024, Ranta et al. 2015). Patients also experience barriers to primary care that may be exacerbated when seeking help for headache or migraine, due to perceived stigma and lack of understanding about migraine from health professionals (McInnernay et al. 2024, Randerson et al. 2025).

### 1.2 Measuring the Economic Impact of Migraine in NZ

Given these opportunities for improving migraine healthcare and the known burden of migraine, information about the economic impact of migraine in NZ could help identify where resources are being lost and where interventions may reduce costs by reducing healthcare use and increasing productivity. Many migraine cost of illness (COI) studies have been published internationally, with estimates of economic impact ranging from around £12 billion in the UK (Martins et al. 2023) and €111 billion in the EU (Linde et al. 2012), with a high proportion of costs related to lost productivity (Sanchez‐del‐Rio et al. 2025), but none have included data from NZ. COI studies of long‐term conditions in NZ have not included migraine or chronic pain (Hogan and Song 2022, Ministry of Health 2009). An analysis of health system costs in NZ due to non‐communicable diseases found that neurological conditions were second only to musculoskeletal conditions in contributing to health system costs (the contribution of migraine was not separately analysed) and recommended more research and policy work for these conditions (Blakely et al. 2019).

A scoping review of migraine COI studies identified a range of methodologies that could be adapted to the NZ context and summarised the type of data needed, including migraine prevalence in NZ, health utilisation and loss of productivity due to migraine and the cost of healthcare items and productivity loss (Imlach et al. 2026). This review noted best practice in migraine COI studies, relating to perspective, types of cost included, costing approach, migraine case definition and data derived from population not patient samples, with the following recommendations:

• Use a migraine case definition based on the International Classification of Headache Disorders (ICHD) (Headache Classification Committee of the International Headache Society (IHS) 2018) (Table 1).
• Base prevalence estimations on population‐based data, as many people with migraine are not identifiable in health data collections which rely both on people seeking healthcare for the condition and receiving an accurate diagnosis.
• A bottom‐up costing approach using population survey data is preferrable to top‐down approaches using administrative data such as health records (Ashina et al. 2021).
• Take a societal perspective, which considers all costs incurred by patients, families, the health system, employers and government.
• Include both direct and indirect costs, as indirect costs are often much greater than direct costs (Linde et al. 2012).
• Include a sufficient range of direct and indirect costs; direct costs are predominantly from healthcare use, including contacts with primary and secondary care (and complementary health services), pharmaceuticals and diagnostic tests; indirect costs are incurred through loss of productivity due to absence from work (absenteeism), reduced efficiency when working with migraine symptoms (presenteeism), reduced employment and impact on unpaid labour.

**TABLE 1:** Classification of migraine.

The International Classification of Headache Disorders (ICHD‐3)^a^ from the International Headache Society define migraine when a patient has experienced at least five attacks that fulfil the following criteria (Headache Classification Committee of the International Headache Society (IHS) 2018):
1. Each headache attack lasts 4 to 72 h (when untreated or unsuccessfully treated).
2. During the headache, the patient has at least two of the following four characteristics: • Unilateral location (one side of the head) • Pulsating quality (throbbing pain) • Moderate to severe intensity (interferes with daily activities) • Aggravation by routine physical activity (e.g. walking or climbing stairs)
3. During the headache, at least one of the following occurs: • Nausea and/or vomiting • Photophobia (sensitivity to light) and phonophobia (sensitivity to sound)
4. The headache is not better accounted for by another diagnosis in the ICHD‐3 classification

a ICHD first developed in 1988, updated in 2004 (version 2) and 2018 (version 3, following a beta version in 2013). Versions 2 and 3 made no significant changes to the classification of migraine.

Estimates of prevalence and cost also need to include key demographics, particularly age and gender, as migraine varies by these characteristics. In the NZ context, they also need to include ethnicity, so the health and social impact of migraine on Māori can be measured. This is an obligation under Te Tiriti o Waitangi and is embedded in health legislation (Pae Ora Act 2022), which demands accountability for Māori health equity from the whole health system. In NZ, both Māori and Pacific peoples experience worse health outcomes than the European population, due to many factors such as differential access to economic resources and healthcare, layers of discrimination and, for Māori, the ongoing impacts of colonisation. Although there is limited information on Māori and Pacific peoples’ experience of migraine, it is likely to be affected by these same factors.

The next step in undertaking a robust, comprehensive and NZ‐specific migraine COI is to identify existing data that could be used and what new data might be needed. We undertook a review of primary data sources in NZ that could be used for this and potentially other research projects on migraine in NZ. The review aimed to evaluate existing datasets against best practice recommendations, identify data deficiencies and gaps and recommend how these may be filled.

## 2 Methods

This review adapted methods developed for data source reviews by the Centre of Methods and Policy Application in the Social Sciences (COMPASS) at the University of Auckland (Boven et al. 2023, Underwood et al. 2025).

### 2.1 Identification of Data Sources

The first stage involved identifying relevant NZ data sources that contained information on migraine prevalence, direct and indirect costs from migraine.

We reviewed datasets in the Global Burden of Disease (GBD) study (NZ sources) (Global Burden of Disease Collaborative Network 2025b), Stats NZ surveys, national administrative data collections from Te Whatu Ora/Health NZ (responsible for the publicly funded health system of NZ), Ministry of Social Development (administrator of sickness, disability and unemployment benefits), websites of Primary Health Organisations (PHOs) (primary care data not collected centrally by Te Whatu Ora, as most primary care practices are private businesses), Accident Compensation Corporation (national compulsory accident and injury insurance provider), Hato Hone St John and Wellington Free Ambulance (national emergency paramedic and ambulance services), sources listed in previous COMPASS reports and key NZ longitudinal and birth cohort studies. Data sources were included if they contained information relating to migraine or could be linked to information on migraine. We also reviewed datasets included in the Integrated Data Infrastructure (IDI) (Stats NZ 2026), a large database of linked, deidentified administrative and survey data collected from government agencies, Stats NZ and other sources, including information on health, welfare, tax and employment. For cost information, we also searched the websites of the Ministry of Health and Pharmac (agency responsible for funding of medicines in NZ).

### 2.2 Literature Searches

We conducted focused literature reviews for additional studies on migraine prevalence, health service use and productivity loss in people with migraine in NZ, searching for original research published in English. We searched Medline, Embase and Scopus from database inception until 9 February 2026 (search strategies in Supplementary Material A). ST undertook the initial screening of articles, excluding those that did not include migraine or were not NZ based, with articles identified for possible inclusion then reviewed by FI and ST. We also reviewed reference lists and citations of studies included in the final evaluation.

For prevalence studies, we also reviewed studies from NZ included in a recent narrative review of all published studies on headache prevalence up until 2020 (Stovner et al. 2022). Only population‐based studies of migraine were included in the final analysis, as recommended by the Global Campaign against Headache (Stovner et al. 2014).

For health service use, we focused on healthcare services most commonly included in migraine COI studies: hospitalisations, emergency department (ED), outpatient and primary care visits and pharmaceuticals. We also noted information on other costs that would be useful to include such as complementary or alternative therapies and non‐health costs of transport to healthcare and receipt of welfare benefits.

### 2.3 Data Source Description

Once data sources were identified, the second stage of the review was to characterise the sources and data they contained according to type of data collection (e.g. cross‐sectional survey, longitudinal, administrative dataset, one‐off collection or repeated); type of information (e.g. lifetime prevalence, 1‐year prevalence, health service use, cost, labour force participation, lost productivity); nature of the sample (population, sample size); ownership and accessibility of data (e.g. available online, by application, through the IDI, not available); sample characteristics (e.g. age, gender, ethnicity); definition of migraine (e.g. self‐report, doctor‐diagnosed, using ICHD classification, using ICD‐10 coding, inclusion of subtypes of migraine); and whether able to be linked to other datasets in the IDI.

### 2.4 Data Source Evaluation

The final stage of the review was to evaluate the data sources. For prevalence studies, we used criteria developed by the Global Campaign against Headache (Stovner et al. 2014) to assess study quality, based on ratings of sampled population, sampling method, number of respondents, participation rate, access, validation of diagnostic instrument, diagnostic criteria and prevalence time frame, with possible scores of −27 to + 32. We added criteria to assess adequate representation of Māori and Pacific people and the availability of datasets for additional analysis and linkage to other data sources.

For data sources on direct and indirect costs, we developed evaluation criteria of relevance (contained information related to people with migraine), population‐based (or able to be linked to population‐based data), accessibility (data likely available to researchers on reasonable request and amenable to further analysis) and information on key demographics (age, gender and ethnicity). For data sources on direct and indirect costs, we reviewed sources that met at least one of these criteria. Although sources that were not ‘relevant’ would not be usable for a COI study, we included those that might have future potential, if migraine or additional information could be added.

## 3 Results

### 3.1 Prevalence Data

From the literature searches, review of datasets, reference lists and citations, we identified 15 studies potentially containing NZ migraine prevalence information for further screening (Supplementary Material B: Data sources screened). Four publications referred to the same data source; two publications were excluded for not being population‐based samples, and one study was excluded as it reported on prevalence of headache but not migraine specifically, leaving nine studies for evaluation (Table 2). These included a one‐off regional cross‐sectional survey, three waves of a national cross‐sectional survey, three longitudinal studies (one birth cohort, one prospective case control study, one fixed panel survey) and two global modelling studies.

**TABLE 2:** Estimates of migraine prevalence in NZ.

Year of publication	Year of data collection	Prevalence time frame	Sample size	Age (years)	Estimate	Data source	Study description	Migraine definition
1993 (Thomson, white and West 1993)	1990	Lifetime	922	17–70 years	22.1% (*n* = 204) Estimates by gender and ethnicity (Māori, Pacific, European), age, migraine with and without aura	Telephone survey (random digit dialling)	Cross‐sectional survey in South Auckland to assess migraine prevalence in Māori, Pacific and European groups, using a sample frame to give roughly equal numbers across ethnicity and gender	Respondents asked if they had ever suffered from bad headaches; then asked about aura and features of migraine based on ICHD‐1 criteria
2002 (Waldie and Poulton 2002)	1998/1999	1 year	979	26	11.7% (*n* = 114; 72 migraine only, 42 with migraine and tension‐ type headache)	Dunedin Multidisciplinary Health and Development Study	Longitudinal birth cohort study, including children born in Dunedin between April 1972 and March 1973. Not representative of NZ population.	Participants were asked if they had ‘frequent headaches in the past 12 months, lasting from 30 min to 7 days.’ Subsequent questions adapted from ICHD‐1 criteria
2014 (Waldie et al. 2014)	2008/2010	1 year	617	11	10.5% (*n* = 65 with migraine)	Auckland Birthweight Collaborative (ABC) Study	Longitudinal prospective case control study comparing babies that are small for gestational age born at term with those appropriate for gestational age Eligible babies born in Waitemata or Auckland from 1995 to 1996 and Auckland from 1996 to 1997. Only NZ European children included in analysis	Children who indicated they suffered from headache for 30 min or more in the past year were asked about the nature, duration and severity of the pain based on ICHD‐3 (beta) criteria (actual questions not provided)
2008 (Carter, Hayward and Richardson 2008) (report only)	2004/2005	Lifetime	20,000 adults	All ages	13.4% (overall, unadjusted, *n* = 2435) Estimates for age, gender, ethnicity and multiple measures of socioeconomic position	Survey of Family, Income and Employment (SoFIE)	Longitudinal panel survey, representative of national population. Health module asked in 2004/2005 2006/2007, 2008/2009 Includes data on age, gender, ethnicity, area deprivation, health region/district	As part of the health module each respondent was asked ‘have you ever been told by a doctor that you had’: Migraines [sic] (and other diseases). If a respondent answered ‘Yes’, they were not asked in following waves.
2008 (Ministry of Health 2008) (report only)	2006/2007	Lifetime	12,488 adults	15 years and over	9.5% (overall, unadjusted, weighted to represent 296,100 adults) Estimates by gender and ethnicity	New Zealand Health Survey	Cross‐sectional nationally representative survey Not included in long term time trend (published as web tool online) as methodological differences from subsequent surveys	As part of the long‐term health conditions module, respondents were asked ‘have you ever been told by a doctor that you had’ and shown a list of conditions on a showcard that included migraine
2022 (data release only) (Migraine Foundation Aotearoa New Zealand 2022)	2013/2014	Lifetime	9717 adults	15 years and over	15.7% (overall, weighted to represent 560,000 adults) Estimates for age, gender, ethnicity	New Zealand Health Survey	Cross‐sectional nationally representative survey Includes data on age, gender, ethnicity, area deprivation, health region/district	Have you ever been told by a doctor that you have migraines [sic]
2026 (Ministry of Health 2026)	2023/2024	Lifetime; migraine symptoms in last 3 months	9719 adults	15 years and over	17% migraine diagnosis (overall, weighted to represent 730,000 adults); 15% positive ID, migraine (628,000 adults) Estimates for age, gender, ethnicity, disability status, area deprivation	New Zealand Health Survey	Cross sectional nationally representative survey Includes data on age, gender, ethnicity, area deprivation, health region/district, disability	Have you ever been told by a doctor that you have migraines [sic]; ID‐Migraine screening tool, which asks about headache in the last 3 months then whether headache limited activities, presence of nausea and light sensitivity (migraine symptoms)
2025 (online tool) (Global Burden of Disease Collaborative Network 2025a)	2023 (and previous years)	1 year	Model based	All ages	15% (age standardised, representing 733,199 people) Estimates for age and gender only	Global Burden of Disease Study 2023	Bayesian models used to estimate prevalence across time, age and sex. Source data include population surveys from 51 countries (Steinmetz et al. 2024)	Based on surveys that measure migraine attacks fulfilling ICHD criteria (at least one in the past 12 months)
2025 (Global Burden of Disease Collaborative Network 2025c)	2021 (and previous years)	1 year	Model based	All ages	15% (age standardised, representing 124,075 people) Estimates for age, gender, Māori and non‐Māori	Global Burden of Disease Study 2021	As above. Māori and non‐Māori population numbers based on resident population data from census data and estimates from Stats NZ (Ministry of Health 2025)	As above

Most studies provided estimates for a range of age groups, with several including children (Global Burden of Disease Collaborative Network 2025a, Carter et al. 2008) and ethnicity (Global Burden of Disease Collaborative Network 2025a, Carter et al. 2008, Thomson et al. 1993). None were able to estimate prevalence of chronic migraine or any other measures of migraine severity. Only one study differentiated types of migraine, estimating prevalence of migraine with and without aura (Thomson et al. 1993).

#### 3.1.1 Regional Studies

Two studies estimated migraine prevalence at specific ages in different regions of NZ. The first used data from the Dunedin Multidisciplinary Health and Development Study (Dunedin Study), a birth cohort that reported 1‐year migraine prevalence at age 26 (Waldie and Poulton 2002). This was also listed in the data input sources used to estimate migraine prevalence in NZ by the GBD 2023 Study (Global Burden of Disease Collaborative Network 2025a). The same data were included in several other publications (Waldie 2001, Waldie et al. 2008, Waldie and Poulton 2002). Questions were adapted from the 1988 ICHD (Waldie et al. 2008) but did not met current best practice. A screening question asked participants if they had experienced frequent headaches lasting 30 min to 7 days in the past 12 months, whereas a neutral screening question is now recommended (Stovner et al. 2014). Participants were asked a series of yes/no questions about their experience of headache which did not strictly correlate to the ICHD classification criteria (Waldie 2001). Hence, the migraine prevalence of 11.7% from this study may be an underestimate.

Another prospective study from Auckland estimated 1‐year migraine prevalence in children aged 11 (Waldie et al. 2014). This was only reported for NZ European children, but found a similar prevalence (10.5%) to that from a systematic review on migraine in children and adolescents (11%) (Onofri et al. 2023). Details of the questions about migraine were not given but stated to be based on the ICHD.

#### 3.1.2 National Studies

Several national population‐based surveys provided estimates of migraine prevalence, including the Survey of Family, Income and Employment (SoFIE) (Carter et al. 2010) and the NZ Health Survey, which asked about migraine in 2006/2007, 2013/2014 and 2023/2024. The methodology of the 2006/2007 survey has been superseded, and results from this are not considered reliable. The results from the 2013/2014 study were not formally published but released after request from one of the authors (FI) (Migraine Foundation Aotearoa New Zealand 2022). All of these surveys are available on application through the IDI, include information about other health conditions and can be linked to other datasets that include additional health and sociodemographic data. However, the case definition of migraine in these surveys was not based on the ICHD criteria, but on self‐reported ‘doctor‐diagnosed’ migraine. These studies may underestimate the true prevalence of migraine, by missing people with undiagnosed migraine who have not sought medical care for migraine or have not been accurately diagnosed. However, the 2023/2024 NZHS also screened for migraine symptoms using the ID‐Migraine tool, which has been validated in multiple countries and settings (sensitivity of 85% and specificity of 76%) (Cousins et al. 2011) and provides an estimate of undiagnosed migraine within this sample.

For all of these studies, the quality score rating was at least ‘acceptable’, with the main limitation being the lack of validation of the diagnostic instrument (Supplementary Material C: Evaluation of migraine prevalence data).

#### 3.1.3 GBD Studies

The final two results from the GBD studies could not be evaluated, as the prevalence estimates are derived from complex modelling that combines meta‐analysis and regression, using a range of inputs. From a published analysis of neurological conditions from GBD 2021, global estimates of migraine prevalence were based on 148 prevalence, 4 incidence and 7 other studies from 51 countries, which used ICHD‐3 criteria to classify migraine (Steinmetz et al. 2024). The online data sources tool for GBD 2023 included 166 citations for migraine (Global Burden of Disease Collaborative Network 2025b). This modelling approach creates estimates for gender and age even in countries where primary data are limited (Hay et al. 2025). In 2025, estimates of disease burden for Māori were published using GBD 2021 data (Ministry of Health 2025), which provided migraine prevalence estimates for Māori and non‐Māori, by gender and age.

### 3.2 Data on Direct Costs

From the literature searches, review of datasets, reference lists and citations, we identified 16 studies potentially containing information on migraine direct costs and a range of data collections (Supplementary Material B). Table 3 lists data sources that either contain some information on migraine‐specific healthcare use or other direct cost information that could be linked to population‐based migraine prevalence.

**TABLE 3:** Data sources for direct costs for migraine in NZ.

Direct costs	Potential data source—service use information	Migraine definition	** Evaluation** a
**Core healthcare services (for migraine cost of illness study)**			
Hospitalisations (public and private)	National Minimum Dataset (NMDS), national collection of all public hospital discharges for inpatients and day patients (from 1998) and privately funded discharges, including length of stay (from 2001), linked to costing information available from the Ministry of Health	ICD‐9 (346.xx codes) or ICD‐10 (G43.xx codes)	Rel, Link, Acc, Char
Emergency department (ED) visits	National Non‐admitted Patient Collection (NNPAC), national collection of ED visits lasting <3 h and publicly funded outpatient attendances (from 2007)	No diagnostic information in NNPAC	Link, Acc
	Migraine in Aotearoa NZ (MiANZ) survey (2022) asked whether seen in ED in last 12 months or >12 months ago (patient not population‐based survey)	Positive ID‐Migraine screen or self‐reported doctor diagnosed migraine	Rel, Acc
	Headache in Emergency Departments (HEAD) study (2019) (Wijeratne et al. 2022, Kelly et al. 2021, Chu et al. 2023, Chu et al. 2022, Pellatt et al. 2021)	Migraine diagnosis on ED discharge	Rel
	Audit of pain management practices in paediatric EDs (Herd et al. 2009)	Migraine diagnosis on ED discharge	Rel
	NZ Health Survey asked about visits to ED at a public hospital in the past 12 months (but not reason for visits)	Doctor‐diagnosed migraine in 2006/2007, 2013/2014 and 2023/2024	Pop, Acc, Char
Specialist (outpatient) visits	NNPAC, national collection of emergency and outpatient attendances (from 2007)	No diagnostic information in NNPAC	Acc, Link
	MiANZ survey (2022) asked whether neurologist/pain specialist seen for migraine in last 12 months or >12 months ago	Positive ID‐Migraine screen or self‐reported doctor diagnosed migraine	Rel, Acc
Primary care visits	Data from 13 Primary Health Organisations (PHOs)	READ or SNOMED codes	Rel, Char
	PHO enrolment data	No diagnostic information and not all visits are recorded	Acc, Link
	MiANZ survey (2022) asked whether general practitioner seen for migraine in last 12 months or >12 months ago	Positive ID‐Migraine screen or self‐reported doctor diagnosed migraine	Rel, Acc
	NZ Health Survey asked about visits to primary care in the past 12 months (but not reason for visits)	Doctor‐diagnosed migraine in 2006/2007, 2013/2014 and 2023/2024	Pop, Acc, Char
Prescription pharmaceuticals	Pharmaceutical Collection (PHARMS) records claims from community‐based pharmacists for dispensing of all government‐funded prescription medications (from 2005)	Dispensing of migraine‐specific medication (e.g. triptans, pizotifen)	Rel, Link, Acc Char
	MiANZ survey 2022 (current use of acute and preventive treatments, frequency of acute medication use)	Positive ID‐Migraine screen or self‐reported doctor diagnosed migraine	Rel, Acc
Diagnostic tests (particularly head scans)	Laboratory Claims Collection contains laboratory test information	No results or reason for testing included in laboratory data	Link, Acc
	No records or collections of imaging tests		
**‘Nice to have’ healthcare services**			
Ambulance	Aotearoa New Zealand Paramedic Care Collection includes road‐based ambulance (emergency medical service) attendances and transfers for St John/Hato Hone and Wellington Free Ambulance (from 2019)	Ambulance personnel clinical impression	Link, Acc, Char
Over‐the‐counter pharmaceuticals	MiANZ survey – see above under Prescription pharmaceuticals		
Complementary therapies	MiANZ survey (2022) asked about other health practitioners seen in last 12 months or >12 months ago, current use of supplements and complementary therapies	Positive ID‐Migraine screen or self‐reported doctor diagnosed migraine	Rel, Acc
Post head injury migraine	Accident Compensation Corporation (ACC) data on claims for treatment and income compensation after an injury claim (migraine can occur after head injury)	Migraine diagnosed and attributed to the injury by a medial practitioner	Rel, Link, Acc, Char
** Non‐healthcare direct costs**			
Social welfare benefits	Benefits dynamics data from the Ministry of Social Development records receipt of unemployment and disability benefits for working age people (from 1990)	Migraine can be listed as an incapacity (limiting ability to work) by a medical practitioner	Rel, Link, Acc, Char
	Accident Compensation Corporation (ACC) also provides benefits (see above)		
	MiANZ survey (2022) asked about benefits and work‐related difficulties due to migraine, which included ‘cannot work’ but didn’t ask specifically about receipt of benefits because of migraine	Positive ID‐Migraine screen or self‐reported doctor diagnosed migraine	Acc
Travel to healthcare visits	MiANZ survey (2022) asked about reasons people were unable to see a health professional for migraine, which included ‘no transport’	Positive ID‐Migraine screen or self‐reported doctor diagnosed migraine	Rel, Acc
Care of a dependent	MiANZ survey (2022) asked about reasons people were unable to see a health professional for migraine, which included ‘could not arrange childcare or care for a dependent’	Positive ID‐Migraine screen or self‐reported doctor diagnosed migraine	Rel, Acc

a Rel = relevant (contains relevant direct cost information on people with migraine), Pop = uses population‐based data to identify people with migraine, Link = linkable to a population‐based migraine prevalence survey, Acc = accessible (data available to researchers), Char = includes key characteristics of people with migraine (age, gender and ethnicity).

#### 3.2.1 Hospitalisations

The primary source for migraine‐related hospitalisation is the National Minimum Dataset (NMDS). We did not find any population‐based survey data that asked about hospitalisations for migraine. However, data from the NZHS and SoFIE (migraine prevalence surveys) could be linked to the NMDS, which would identify previous migraine‐related hospitalisations in people with migraine in these survey samples.

#### 3.2.2 ED and Outpatient Visits

We found limited information on ED attendances and outpatient visits at public hospitals for migraine. Although hospital admissions from ED are included in the NMDS, short‐stay ED and outpatient visits recorded in the National Non‐admitted Patient Collection (NNPAC) do not include diagnostic information and therefore cannot identify migraine presentations or migraine treatment. The NNPAC could be linked to migraine prevalence surveys to identify people with migraine attending ED or outpatients, but this would be for any cause, not migraine specifically.

Migraine Foundation Aotearoa NZ made an Official Information Act (OIA) request in 2023 to Te Whatu Ora asking about the number of referrals to outpatient neurology clinics in public hospitals for headache or migraine (Imlach 2024). However, this information was only available for some regions and excluded large hospitals in Auckland and Waikato. Due to differences in reporting, only four hospitals throughout the country were able to provide information on how many headache or migraine referrals were seen in person over the previous 12 months (ranging from 4% to 43%). Te Whatu Ora was formed in 2022 to replace 22 district health boards and provide a national health system that minimised regional variation. Standardised health data collection across the public hospitals may improve in the future.

The Migraine in Aotearoa NZ (MiANZ) survey of 530 people with migraine asked participants about being seen at ED or by a neurologist/pain specialist for migraine in the last 12 months, but this was not a population‐based survey (McInnernay et al. 2024). Conversely, the NZHS, which is a population‐based survey, asked respondents about visits to ED (and primary care) in the past 12 months, but not the reason for these visits, so migraine‐specific healthcare cannot be ascertained.

An international, multicentre study of headache presentations to ED included nine public hospitals from NZ, from which 18% of headache presentations were migraine (*n* = 108) (Wijeratne et al. 2022), but no further NZ‐specific analysis on those with migraine was published. This study estimated that 1% of ED caseload was due to headache presentations (Kelly et al. 2021). An audit of pain management in paediatric EDs included migraine cases from two EDs in NZ but did not report the NZ data separately (Herd et al. 2009). No information on specialist visits for migraine in the private sector was found although in 2024 it was estimated that around 22% of specialist medical workforce hours were spent in private practice (Keene 2025).

#### 3.2.3 Primary Care

The IDI includes a national database on people enrolled in one of the 13 primary care organisations (PHOs) in NZ, which co‐ordinate contracts between primary health services and the government. This can be combined with other datasets to estimate visits to primary care; however, coverage is not complete and there is no corresponding information on diagnosis or treatment. In addition, the PHO dataset only records a maximum of one visit per month (or quarter, in an older version of the data).

Many PHOs undertake analysis of their own primary care data, but these data are not readily available. One analysis of data from a large PHO in Auckland (Mugridge et al. 2026) found a very low prevalence of recorded migraine (5%) in electronic health records and did not report on the number of primary care visits for migraine. The MiANZ survey asked people with migraine about seeing a GP in the last year but not frequency of visits. The government is currently working on a national primary care dataset (Health New Zealand/Te Whatu Ora 2026), but the usefulness of this for a COI study will depend on the quality of the diagnostic data.

#### 3.2.4 Pharmaceuticals

There is partial information on the use of medications to treat migraine. At a national level, the Pharmaceutical Collection (PHARMS) includes dispensed, publicly funded medications but does not include over‐the‐counter medications commonly used to treat migraine attacks such as non‐steroidal anti‐inflammatory drugs. PHARMS will record dispensing of triptans (sumatriptan and rizatriptan are available in NZ), for which the only other indication for use is cluster headache, a rare condition. Sumatriptan tablets can also be purchased over‐the‐counter from a pharmacist, but most people use triptans from their prescriber. Unfunded medications (like the anti‐CGRP migraine medications) are not included in PHARMS.

PHARMS has been used in a study monitoring trends in analgesic use in older people that included ‘anti‐migraine’ medications, based on codes from the Anatomical Therapeutic Chemical (ATC) classification (NO2C code group) (Nishtala et al. 2015). Medications classed as ‘anti‐migraine’ include triptans, pizotifen (used in NZ), ergot derivatives (ergotamine is no longer available in NZ although dihydroergotamine may be given as an infusion in some hospitals) and the unfunded CGRP blockers. PHARMS does not record diagnosis or reason for the medication to be dispensed. Hence, for funded migraine preventive medications not specific to migraine (e.g. anti‐hypertensives, anti‐convulsants, beta blockers, anti‐depressants), there is no way to discriminate between use of a beta blocker for migraine or blood pressure (or both).

As with the NMDS, PHARMS can be linked to migraine prevalence survey data to give a picture of medication use by people with migraine, but this would not definitively establish whether medications are used for migraine or another condition. The MiANZ survey asked about acute and preventive migraine medication use in detail (Imlach and Garrett 2024), but this is a sample with a high proportion of people with chronic and frequent migraine and is not representative of the whole migraine population.

#### 3.2.5 Diagnostic Tests

For laboratory tests, the Laboratory Claims Collection contains information about laboratory tests but no diagnostic information. There are no diagnostic tests for migraine, but head scans are sometimes performed to rule out secondary causes for headache symptoms (Chu et al. 2023). There are no national collections on imaging tests in NZ.

#### 3.2.6 Benefits

An important non‐health direct cost is receipt of social welfare payments due to inability to work (Hogan and Song 2022). In NZ, when an individual applies for a health‐related benefit, they need a medical certificate which indicates which ‘incapacity’ or ‘incapacities’ prevent them from being able to work, as per the assessment of the medical practitioner. Between 1995 and 2007, this information was collected via a paper form with tick boxes, which did not list migraine as a potential ‘incapacity’. Since 2007, applications are mostly made through practice management systems and ask for the main diagnoses in order of impact. Migraine Foundation Aotearoa NZ requested information from the Ministry of Social Development about the number of working age people on benefit with migraine listed as an incapacity in 2022—only 429 people were identified (Migraine Foundation Aotearoa New Zealand 2022). By contrast, in the MiANZ survey, 14% of respondents reporting on the impact of migraine on work (*n* = 68) were unable to work because of migraine (Garrett and Imlach 2024).

#### 3.2.7 Other Services

The MiANZ survey asked about a wide range of supplements and complementary therapies used for migraine (Imlach and Garrett 2025) and also asked about reasons people were unable to see a health professional for migraine, including not having transport or not being able to arrange child care but did not ask how frequently this occurred (McInnernay et al. 2024). National data on ambulance attendances are available and can be linked to NMDS data, but analysis of headache or migraine attendances has not yet been undertaken.

#### 3.2.8 Cost Estimates

In NZ, payment for healthcare is mixed. Secondary care is free but private options exist, which may be paid for out‐of‐pocket by the patient or covered by health insurance. Most primary care is privatised but part‐funded by the government, and visits usually involve a charge to the patient. Medications are funded by the government when these have been approved by Pharmac, the drug funding agency, but prescriptions usually incur an out‐of‐pocket cost from a prescriber's fee and a dispensing fee ($5 per dispensed item).

Pharmac's Cost Resource Manual (Pharmac 2025) provides estimates of health system costs to be used in health technology assessments that could be applied to a cost of migraine study. This includes how to cost pharmaceuticals to include dispensing and administrative fees, primary care and hospital costs. However, these costs are for 2017 or 2018. Current case mix cost weights (for hospital admissions), purchase unit costs (for outpatient and ED visits) and subsidies for pharmaceuticals and laboratory tests can be obtained through IDI datasets. A method to estimate total costs from pharmaceuticals accounting for dispensing fees, subsidies, rebates and the cost of a primary care visit has also been developed (Telfar Barnard 2025), and costing methods for other direct and indirect costs could be adapted from other costing studies using IDI data (Blakely et al. 2019, Blakely et al. 2021, Summers et al. 2023). However, private insurance and other out‐of‐pocket costs are not readily measurable using IDI data.

### 3.3 Data on Indirect Costs

The literature searches returned 10 studies with information about migraine in NZ, all of which had been identified in previous searches or reference/citation checking (Supplementary Material B). Table 4 lists data sources that contain indirect cost information that relates to migraine, could include migraine information in the future or could be linked to population‐based migraine prevalence.

**TABLE 4:** Data sources for indirect costs for migraine in NZ.

Indirect cost	Potential data source—indirect cost information	Migraine definition	**Evaluation** **a**
Lost work productivity	Dunedin Longitudinal Study (Waldie and Poulton 2002) On a scale of 1 to 5, how much have these headaches interfered with your work (1 = very little, 5 = very much)	Headaches in the past 12 months, lasting from 30 min to 7 days; subsequent questions adapted from ICHD‐1	Rel, Pop
Absenteeism	MiANZ survey (2022) asked about days of missed school or work over the last 3 months because of headaches (MIDAS)	Positive ID‐Migraine screen or self‐reported doctor diagnosed migraine	Rel, Acc
Presenteeism	MiANZ survey (2022) asked about work‐related difficulties due to migraine, which included ‘full time work but less than best performance’	Positive ID‐Migraine screen or self‐reported doctor diagnosed migraine	Rel, Acc
Lost unpaid work productivity	MiANZ survey (2022) asked about days where people could not do household work or productivity in household work halved or more over last 3 months because of headaches (MIDAS questions)	Positive ID‐Migraine screen or self‐reported doctor diagnosed migraine	Rel, Acc
	General Social Survey (2023) asked about hours of voluntary work and whether ‘health problems’ prevent a person doing voluntary work, but type of health problem not specified	No migraine data	Acc
Under employment and unemployment	MiANZ survey (2022) asked about labour force status work‐related difficulties due to migraine, which included ‘can only work part time’ and ‘cannot work’	Positive ID‐Migraine screen or self‐reported doctor diagnosed migraine	Rel, Acc
	Household Labour Force Survey asks reasons for not working usual hours or more hours, not looking for work, why left last job, for which ‘own sickness, illness, injury, disability’ is an option (but specific cause not asked for)	No migraine data	Link, Acc
	Dunedin Study (1998/1999) reported on labour force status in people with migraine but not whether unemployment is due to migraine (Waldie and Poulton 2002)	Headaches in the past 12 months; questions adapted from ICHD‐1	Pop
	SoFIE (2004, 2006, 2008) and NZHS (2006/2007, 2013/2014, 2023/2024) collect labour force status but not whether this is due to migraine	Doctor‐diagnosed migraine, ID‐Migraine (NZHS 2023/2024)	Pop, Acc
Early retirement	No data		

a Rel = relevant (contains relevant direct cost information on people with migraine), Pop = uses population‐based data to identify people with migraine, Link = linkable to a population‐based migraine prevalence survey, Acc = accessible (publicly available), Char = includes key characteristics of people with migraine (age, gender, ethnicity).

#### 3.3.1 Absenteeism and Presenteeism at Work

There are no national or regional datasets on employees or workplaces that provide information about absenteeism or presenteeism. We did not identify any workplace studies that estimated lost productivity due to migraine in NZ. Two studies asked about impact of headaches on work in people with migraine. The MiANZ survey estimated absenteeism from work or school due to migraine using the Migraine Disability Assessment Scale (MIDAS), a widely used and validated set of questions that assess headache‐related disability (Stewart et al. 2001). From this survey, around 25% of respondents reported missing school or work on more than 5 days (Garrett and Imlach 2024). The MiANZ survey also directly asked about impact of migraine on work, including not being able to work because of migraine. However, as noted above, this was not a population‐based survey and will overestimate impacts due to an over‐representation of chronic migraine. The Dunedin Study, which was a population‐based longitudinal survey, found that headaches ‘interfered with work’ in 39% of those with migraine, but this question was not specific enough to estimate productivity loss.

#### 3.3.2 Unpaid Work

Other MIDAS questions asked in the MiANZ survey relate to being unable to do household work (unpaid labour). Only one national population‐based survey (the General Social Survey) asked about limitations to voluntary work, including health conditions, but did not collect more specific information that would link limitations to migraine. The Census, Time Use Surveys and Household Labour Force Surveys have also asked about volunteer or unpaid work, using a range of definitions (Ministry for Pacific Peoples 2021), but none of these asked about inability to undertake unpaid work.

#### 3.3.3 Under‐ and Unemployment

The MiANZ survey specifically asked about under‐ and unemployment because of migraine but is not generalisable to the whole population. Several other surveys can estimate under‐ and unemployment rates in people with migraine (NZHS, SoFIE, Dunedin Study), but not whether employment status is directly related to the migraine condition. This is particularly problematic for surveys that estimate lifetime prevalence of migraine and not active or current disease (symptoms in the past year). No data were found on prevalence of early retirement due to migraine.

#### 3.3.4 Cost Estimates

To calculate indirect costs at a national level, inputs on the cost of productivity losses are needed, such as national data on average wages (adjusted for age and gender at a minimum), labour force participation and work hours. Stats NZ provides estimates of employment, median weekly and hourly earnings by age, gender, ethnicity, occupation and other characteristics based on the Household Labour Force Survey (Stats NZ 2025). Calculations could assume an 8‐h work day and 240‐day work year. The value of unpaid work can be estimated using minimum wage or average wage of those in comparable work, also available from Stats NZ.

## 4 Discussion

This review identified a range of data sources that could be used for a NZ migraine COI study but also significant gaps. Migraine prevalence data is adequate but has limitations, with population surveys not using ICHD criteria to define migraine. Despite that, migraine prevalence from these surveys is estimated to be 13–17%, similar to estimates from the GBD study (14%–15%), although international surveys using standardised, high‐quality methods give estimates as high as 25% in 18–65 year olds (Husøy et al. 2025). A costing study could include a range of prevalence estimates to account for this uncertainty.

NZ prevalence data includes estimates by age, gender and ethnicity, with potential for additional analysis to explore associations with comorbid conditions and other confounding factors, such as labour force status, income and unemployment benefits. Survey research in NZ should be adequately powered to measure outcomes for Māori (Reid et al. 2017), and there are several data sources that measure migraine prevalence in Māori. Although it can be informative to estimate the cost of migraine for those with more severe disease (Buse et al. 2020, Kikui et al. 2020, Lipton et al. 2020), there are currently no NZ‐specific population estimates for the prevalence of chronic migraine or data on migraine by severity or frequency of attacks.

### 4.1 Data Gaps for Measurement of Direct and Indirect Costs

For direct costs, there are no population‐based surveys that directly ask about healthcare use because of migraine in people with migraine. Partial all‐cause publicly funded healthcare costs could be measured, but the only measurable migraine‐specific healthcare costs are publicly funded migraine‐related hospitalisations and dispensed anti‐migraine medications, which will miss many medications taken for migraine. No existing datasets can provide estimates for ED, outpatient or primary care visits for migraine specifically. The existing datasets on ED and outpatient hospital visits only cover the public health system, and data on visits to private providers are not reported. Around a third of NZ adults have private health insurance (Ministry of Health 2025), which most commonly covers specialist and hospital care. Even if public health system data were improved to include diagnostic information, this would still leave a gap relating to private neurologist visits.

Lack of laboratory or imaging test data is another gap but less significant as these are not a large part of migraine management. Many international migraine COI studies do not estimate costs of complementary therapies or costs of transport to healthcare, usually due to lack of data, although these may contribute moderately to overall costs (Irimia et al. 2020, Lipton et al. 2013, Amoozegar et al. 2022). These data are also lacking in NZ. The apparent low numbers of people with migraine who receive a benefit deserve further exploration.

The largest data gap is for indirect costs. There are no suitable estimates of absenteeism or presenteeism for migraine in NZ, nor for reduced employment or inability to work because of migraine. The cost of lost productivity for those working unpaid (e.g. in child care, home making or volunteer roles), lost productivity from carers of those with migraine and impact of migraine on education or career progression are not often measured or costed in the international literature (Ashina et al. 2021, Stovner et al. 2014), and NZ also lacks data in these areas.

One data source identified in NZ that is not reported in the international literature is ambulance attendances for migraine. Nearly a quarter of ED presentations for migraine arrive by ambulance (Logan et al. 2022, Chu et al. 2022). Electronic collection of emergency ambulance service data in NZ means that this is a cost that could be estimated if existing data were analysed.

### 4.2 Limitations of Existing Datasets

Although patient‐based administrative datasets contain data on many individuals and are conveniently accessible, many lack diagnostic information and sufficient detail about healthcare use. Since they only count individuals with a coded migraine diagnosis or receiving one of a limited number of migraine‐specific medications, they are not representative of all people with migraine (Stovner et al. 2014). A systematic review of 41 studies using diagnostic and/or treatment codes to identify migraine cases in electronic healthcare records found much lower estimates of prevalence than would be expected in almost all studies and only two attempted any type of validation (Forns et al. 2025). From NZ, a study that used dispensed pharmaceutical data to identify people with migraine found a ‘prevalence’ of only 1.7% (Stanley et al. 2020). In other NZ studies, a cohort of people with a migraine ‘diagnosis’ (defined as the time of first migraine‐related hospitalisation or prescription) included ∼130,000 people aged 25–64 (from 2006 to 2016) (Blakely et al. 2021, Summers et al. 2023), compared to the GBD study estimate of ∼465,000 25–64 year olds with migraine in 2016 (Global Burden of Disease Collaborative Network 2025a).

As well as the issue of under‐identification of people with migraine, administrative datasets often cannot identify migraine‐specific costs but only total costs (e.g. pharmaceuticals dispensed without knowing the disease indication). An approach to this is an excess cost analysis that compares total costs in those with migraine and a matched control group without migraine (Onukwugha et al. 2016). However, this must account for comorbidities (Blakely et al. 2019), particularly mental health and other pain conditions (Stovner et al. 2014), or costs will be overstated (Summers et al. 2023).

For conditions such as migraine, which can and should be managed predominantly in primary care, the lack of robust and integrated primary care data (due to the semi‐private model of primary care in NZ, where most practices are private businesses that receive various amounts of government funding) is the most significant healthcare data gap and creates a dependence on surveys for monitoring the prevalence and impact of these conditions.

### 4.3 Options for a Future Migraine COI Study

There are several options for a future NZ migraine COI study. The first would be to undertake data collection to fill the existing gaps, through either a new population‐based survey or adding extra questions to an existing survey such as the NZHS. The HARDSHIP questionnaire is an example of a validated questionnaire with modules on healthcare resource consumption and societal economic burden that has been used in recent surveys of migraine prevalence and burden (Husøy et al. 2025, Wei et al. 2024, Krishnan et al. 2025). Any new survey should prioritise an accurate case definition of migraine based on ICHD criteria, which is considered the single most important factor in estimating migraine prevalence and cost (Stovner et al. 2022).

Another option would be to explore the utility of administrative health datasets within the IDI, taking into account the limitations noted above, comparing demographics, socioeconomic factors, healthcare use and other outcomes of people with migraine identified in administrative data against people with migraine identified in population‐based survey data. This could identify potential biases in the administrative datasets, estimate their strength and direction and investigate whether adjustment for these biases through modelling and sensitivity analyses is appropriate.

A final option for a NZ study is to review and use international estimates of healthcare use and lost productivity from surveys of people with migraine from similar countries to NZ, such as Australia and the UK (Sanderson et al. 2013, Bloudek et al. 2012). This relies on assumptions that people in NZ have comparable migraine experiences to those in other countries, which is unlikely to be true as all health systems and populations are unique and change over time. However, international estimates could be used as a starting point and tested for credibility through consultation with NZ clinicians, experts and patients, comparison with available data and what is known about healthcare use and productivity for similar chronic conditions in NZ. A range of estimates based on consensus opinion could be developed for use in a NZ‐specific migraine COI model. As for any new survey estimates, international estimates would need to be based on representative population‐based surveys using ICHD criteria to define the migraine sample to minimise risk of misclassification bias.

### 4.4 Conclusion

A migraine COI study in NZ using only currently available data would not meet best practice for a comprehensive and robust analysis, but additional research to fill data gaps is feasible. More complete data on both direct and indirect costs are needed, particularly on lost productivity from migraine, as when considering investments into healthcare, effective treatment will have benefits beyond the health system.

## Funding

This work was supported by the Health Research Council of New Zealand (grant 24/1297/A).

## Conflicts of Interest

The authors declare no conflicts of interest.

## Supporting information

Supplementary Material
