# Supplementary material for: What's Needed for a Migraine Cost‐Of‐Illness Study in Aotearoa New Zealand: Review of Data Sources and Gaps
Source: J R Soc N Z. 2026 May 18;56(3):e70055. doi: 10.1002/snz2.70055 (PMC13182912; doi:10.1002/snz2.70055)
Supplement: Supplementary file 1 — Supplementary Material [file SNZ2-56-e70055-s001.pdf]

Supplementary material A: Search strategies

Prevalence

Ovid MEDLINE(R) ALL <1946 to February 09, 2026>

1 prevalence.mp. or exp Prevalence/
2 epidemiology.mp. or exp Epidemiology/
3 exp Epidemiologic Methods/ or epidemiological methods.mp.
4 morbidity.mp. or exp Morbidity/
5 exp "Global Burden of Disease"/ or burden.mp.
6 migraine.mp. or exp Migraine Disorders/
7 exp Headache/ or headache.mp.
8 exp New Zealand/ or zealand.mp.
9 6 or 7
10 1 or 2 or 3 or 4 or 5
11 8 and 9 and 10
(117 results)

EMBASE - All years <1947-Present with Daily Update>

1 exp prevalence/ or prevalence.mp.
2 epidemiology.mp. or exp epidemiology/
3 exp epidemiological data/
4 exp global disease burden/ or burden.mp.
5 exp morbidity/ or morbidity.mp.
6 1 or 2 or 3 or 4 or 5
7 exp migraine/ or migraine.mp.
8 exp headache/ or headache.mp.
9 7 or 8
10 exp New Zealand/ or zealand.mp.
11 6 and 9 and 10
(263 results)

Scopus

(TITLE-ABS-KEY (migraine OR headache) AND TITLE-ABS-KEY (zealand) AND TITLE-ABS-KEY (prevalence OR epidemiological OR epidemiology OR burden OR morbidity)) AND (LIMIT-TO (LANGUAGE , "English"))
(147 results)

Direct costs

Ovid MEDLINE(R) ALL <1946 to February 09, 2026>

1 primary care.mp. or exp Primary Health Care/
2 secondary care.mp. or exp Secondary Care/
3 exp Tertiary Healthcare/ or tertiary care.mp.
4 exp Pharmaceutical Preparations/ or pharmaceutical.mp.
5 exp "Appointments and Schedules"/ or appointment.mp.
6 exp "Costs and Cost Analysis"/ or cost.mp.

7 exp Health Services/
8 exp Hospitalization/ or hospitali$.mp.
9 exp General Practice/
10 exp "Delivery of Health Care"/
11 exp Emergency Service, Hospital/
12 exp Emergency Medicine/
13 exp Neurology/
14 1 or 2 or 3 or 4 or 5 or 6 or 7 or 8 or 9 or 10 or 11 or 12 or 13
15 exp Migraine Disorders/ or migraine.mp.
16 14 and 15
17 exp New Zealand/ or zealand.mp.
18 16 and 17
(19 results)

EMBASE - All years <1947-Present with Daily Update>
1 primary care.mp. or exp primary medical care/
2 exp secondary health care/ or secondary care.mp.
3 exp tertiary health care/ or tertiary care.mp.
4 exp drug therapy/ or drug therapy.mp.
5 cost.mp. or exp "health care cost"/
6 exp health service/
7 hospitali$.mp. or exp hospitalization/
8 emergency.mp. or exp emergency care/
9 general practice.mp. or exp general practice/
10 neurology.mp. or exp neurology/
11 1 or 2 or 3 or 4 or 5 or 6 or 7 or 8 or 9 or 10
12 exp migraine/ or migraine.mp.
13 11 and 12
14 exp New Zealand/ or zealand.mp.
15 13 and 14
(97 results)

Scopus
(TITLE-ABS-KEY (migraine) AND TITLE-ABS-KEY (zealand) AND TITLE-ABS-KEY (cost OR healthcare OR primary OR secondary OR tertiary OR referral OR hospital OR emergency OR pharmaceutical OR medicine OR medication OR neurology)) AND (LIMIT-TO (LANGUAGE , "English"))
(57 results)

Indirect costs

Ovid MEDLINE(R) ALL <1946 to February 09, 2026>
1 exp Absenteeism/ or absenteeism.mp.
2 productivity.mp. or exp Efficiency/
3 exp Sick Leave/ or medical leave.mp.
4 presenteeism.mp. or exp Presenteeism/
5 work.mp. or exp Work/
6 disab*.mp.

7 impairment.mp.
8 labour.mp.
9 1 or 2 or 3 or 4 or 5 or 6 or 7 or 8
10 migraine.mp. or exp Migraine Disorders/
11 exp Headache/ or headache.mp.
12 10 or 11
13 9 and 12
14 zealand.mp. or exp New Zealand/
15 aotearoa.mp.
16 14 or 15
17 13 and 16
(19 results)

EMBASE - All years <1947-Present with Daily Update>
1 exp absenteeism/ or absenteeism.mp.
2 exp productivity/ or productivity.mp.
3 exp medical leave/ or sick leave.mp.
4 exp presenteeism/ or presenteeism.mp.
5 work.mp. or exp work/
6 labour.mp. or exp labor/
7 disability.mp. or exp disability/
8 impairment.mp.
9 1 or 2 or 3 or 4 or 5 or 6 or 7 or 8
10 migraine.mp. or exp migraine/
11 exp headache/ or headache.mp.
12 10 or 11
13 exp New Zealand/ or zealand.mp.
14 aotearoa.mp.
15 13 or 14
16 9 and 12 and 15
(130 results)

Scopus
(TITLE-ABS-KEY (migraine OR headache) AND TITLE-ABS-KEY (zealand) AND TITLE-ABS-KEY (absenteeism OR presenteeism OR productivity OR disability OR work OR labour OR labor OR efficiency OR (medical AND leave) OR (sick AND leave))) AND (LIMIT-TO (LANGUAGE , "English"))
(76 results)

Supplementary material B: List of data sources screened

Prevalence

Potential data source/reference	Outcome	Reason
Thomson, A N, GE. White, and R. West. 1993. “The Prevalence of Bad Headaches Including Migraine in a Multiethnic Community.” *New Zealand Medical Journal* 106 (967): 477–80	Include in review	
Waldie, Karen E., and Richie Poulton. 2002. “The Burden of Illness Associated With Headache Disorders Among Young Adults in a Representative Cohort Study.” *Headache: The Journal of Head and Face Pain* 42 (7): 612–19. https://doi.org/10.1046/j.1526-4610.2002.02148.x.	Include in review	
Waldie, K. E., and R. Poulton. 2002. “Physical and Psychological Correlates of Primary Headache in Young Adulthood: A 26 Year Longitudinal Study.” *Journal of Neurology, Neurosurgery, and Psychiatry* (England) 72 (1): 86–92. https://doi.org/10.1136/jnnp.72.1.86.	Reference	Duplicate
Waldie, Karen E. 2001. “Childhood Headache, Stress in Adolescence, and Primary Headache in Young Adulthood: A Longitudinal Cohort Study.” *Headache: The Journal of Head and Face Pain* 41 (1): 1–10. https://doi.org/10.1046/j.1526-4610.2001.111006001.x.	Reference	Duplicate
Waldie, K.E., McGee, R., Reeder, A.I. and Poulton, R. (2008), Associations Between Frequent Headaches, Persistent Smoking, and Attempts to Quit. Headache: The Journal of Head and Face Pain, 48: 545-552.	Reference	Duplicate
Waldie, Karen E., John MD Thompson, Yasmine Mia, Rinki Murphy, Clare Wall, and Edwin A. Mitchell. 2014. “Risk Factors for Migraine and Tension-Type Headache in 11 Year Old Children.” *The Journal of Headache and Pain* 15 (1): 60. https://doi.org/10.1186/1129-2377-15-60.	Include in review	
Carter, K, M Hayward, and K Richardson. 2008. *SoFIE-Health Baseline Report: Study Design and Associations of Social Factors and Health in Waves 1 to 3. SoFIE-Health Report 2*. University of Otago, Wellington.	Include in review	
Ministry of Health,. 2008. *A Portrait of Health. Key Results of the 2006/07 New Zealand Health Survey*. Ministry of Health.	Include in review	
Migraine Foundation Aotearoa New Zealand. 2022. “New Zealand Health Survey Data - a Decade to Be Released.” *Https://Migrainefoundation.Org.Nz/New-Zealand-Health-Survey-Data-a-Decade-to-Be-Released/*, October. https://migrainefoundation.org.nz/new-zealand-health-survey-data-a-decade-to-be-released/	Include in review	
Ministry of Health 2023/24 New Zealand Health Survey[77]	Include in review	
Global Burden of Disease Collaborative Network. 2025. *Global Burden of Disease Study 2023 (GBD 2023) Results*. IHME, University of Washington. https://vizhub.healthdata.org/gbd-results/.	Include in review	
Global Burden of Disease Collaborative Network. 2025. *Global Burden of Disease Study 2021 (GBD 2021) Results*. IHME, University of Washington. https://vizhub.healthdata.org/gbd-results/.	Include in review	

Paulin, Judith M., Hendrika J. Waal-Manning, F. Olaf Simpson, and Robert G. Knight. 1985. "The Prevalence of Headache in a Small New Zealand Town." *Headache: The Journal of Head and Face Pain* 25 (3): 147–51. https://doi.org/10.1111/j.1526-4610.1985.hed2503147.x	Exclude	Headache only
Garrett, Susan, and Fiona Imlach. 2024. "The Impact of Living with Migraine Disease in Aotearoa New Zealand." *New Zealand Medical Journal* 137 (1592): 54–76 (Migraine in Aotearoa NZ Survey)	Exclude	Not population based
Stanley, J. , Doughty, R. & Sarfati, D. (2020). A Pharmaceutical Dispensing–based Index of Mortality Risk From Long-term Conditions Performed as well as Hospital Record–based Indices. *Medical Care, 58* (2), e9-e16	Exclude	Not population based
Chan J.K. & Consedine N.S. (2014). Negative affectivity, emotion regulation, and coping in migraine and probable migraine: a New Zealand case-control study. International Journal of Behavioral Medicine, 21(5), 851-860	Exclude	Not population based (case control study)
Carter KN, Anderson N, Jamrozik K, Hankey G, Anderson CS; Australasian Co-operative Research on Subarachnoid Haemorrhage Study (ACROSS) Group. Migraine and risk of subarachnoid haemorrhage: a population-based case-control study. J Clin Neurosci. 2005 Jun;12(5):534-7	Exclude	Not population based (case control study)
Wilsmore BR; Grunstein RR; Fransen M; Woodward M; Norton R; Ameratunga S. Sleep habits, insomnia, and daytime sleepiness in a large and healthy community-based sample of New Zealanders. J Clin Sleep Med 2013;9(6):559-566.	Exclude	Asked about migraine but no reported results; not population based

Other surveys reviewed:

- Christchurch Health and Development Study
- Growing Up in New Zealand Study
- General Social Survey
- Household Disability Survey
- Youth2000 Survey Series
- Youth, Health & Wellbeing Survey
- Te Kupenga: Post censal survey of Māori well being
- New Zealand Health, Work and Retirement Study (and Longitudinal Study of Ageing)

Direct costs

Datasets/studies reviewed

- Accident Compensation Corporation
- Pharmaceutical Collection (PHARMS)
- National Minimum Dataset (NMDS)
- Benefit dynamics data

- Aotearoa New Zealand Paramedic Care Collection
- National Non-Admitted Patients Collection (NNPAC)
- Laboratory Claims Collection
- Primary Health Organisation enrolment
- InterRAI
- National Needs Assessment and Service Coordination Information System (SOCRATES)
- Survey of Family, Income and Employment (SoFIE)
- Headache in Emergency Departments (HEAD) study
- New Zealand Health Survey
- New Zealand Health Survey
- PRIMHD (Progamme for the Integration of mental health data)
- Migraine in Aotearoa NZ Survey 2022
- National Mortality Collection
- National Maternity Collection
- General Medical Subsidy Collection

Studies screened from literature searches:

Article	Data source
Blakely T., Sigglekow F., Irfan M., Mizdrak A., Dieleman J., Bablani L., Wilson N. (2021). Disease-related income and economic productivity loss in New Zealand: A longitudinal analysis of linked individual-level data. PLoS Medicine, 18(11)	Administrative data
Blakely T, Kvizhinadze G, Atkinson J, Dieleman J, Clarke P. (2019). Health system costs for individual and comorbid noncommunicable diseases: An analysis of publicly funded health events from New Zealand. PLoS Medicine, 16(1):e1002716	Administrative data
Chu K., Kelly A.-M., Kinnear F., Keijzers G. & Kamona S. (2022). Primary headache drug treatment in emergency departments in Australia and New Zealand. Medical Journal of Australia, 217(7), 366-367	ED study (HEAD study)
Herd D.W., Babl F.E., Gilhotra Y. & Huckson S. (2009). Pain management practices in paediatric emergency departments in Australia and New Zealand: A clinical and organizational audit by national health and medical research council's national institute of clinical studies and paediatric research in emergency departments international collaborative: Paediatric emergency medicine. EMA - Emergency Medicine Australasia, 21(3), 210-221	Pediatric ED audit
Imlach F. & Garrett S. (2024). Use of medications for migraine in Aotearoa New Zealand. New Zealand Medical Journal, 137(1599), 65-87	Patient survey (Migraine in Aotearoa NZ)
Imlach F. & Garrett S. (2025). Use of Non-Pharmacological Approaches for Migraine Treatment: Results from the Migraine in Aotearoa New Zealand Survey. Journal of Clinical Medicine, 14(12)	Patient survey (Migraine in Aotearoa NZ)
Kelly A.M., Kuan W.S., Keijzers G., Karamercan M.A., Wijeratne T., Graham C.A., Russell K. (2021). Epidemiology, investigation, management, and outcome of headache in emergency departments (HEAD study)-A multinational observational study. Headache, 61(10), 1539-1552	ED study (HEAD study)
McInnarney B., Imlach F., Kennedy J. & Garrett S.M. (2024). Patient perceptions of barriers to effective migraine management in Aotearoa New Zealand. Journal of Primary Health Care, 16(4), 347-356	Patient survey (Migraine in Aotearoa NZ)
Nishtala, P.S., Oh, S., Kim, D. *et al.* Analgesic Medicine Utilization in Older People in New Zealand from 2005 to 2013. (2015). Drugs - Real World Outcomes 2, 177–185	Administrative data

Randerson J., Imlach F., Kennedy J. & Garrett S. (2025). Primary care clinicians' perspectives on migraine management in Aotearoa New Zealand: a qualitative study. Journal of Primary Health Care	Qualitative study of primary care clinicians – no healthcare use
Stanley J., Doughty R.N. & Sarfati D. (2020). A Pharmaceutical Dispensing-based Index of Mortality Risk from Long-term Conditions Performed as well as Hospital Record-based Indices. Medical Care, 58(2), E9-E16.	Administrative data
Thomson A.N., White G.E. & West R. (1993). The prevalence of bad headaches including migraine in a multiethnic community. The New Zealand medical journal, 106(967), 477-480	South Auckland survey – no healthcare use
Waldie K.E. (2001). Childhood headache, stress in adolescence, and primary headache in young adulthood: A longitudinal cohort study. Headache, 41(1), 1-10	Dunedin Study (birth cohort) – no healthcare use
Waldie KE & Poulton R (2002). The burden of illness associated with headache disorders among young adults in a representative cohort study. Headache, 42(7), 612-9	Dunedin Study (birth cohort) – no healthcare use
Waldie K.E., Thompson J.M.D., Mia Y., Murphy R., Wall C. & Mitchell E.A. (2014). Risk factors for migraine and tension-type headache in 11 year old children. Journal of Headache and Pain, 15(1),	Dunedin Study (birth cohort) – no healthcare use
Wijeratne T., Kuan W.S., Kelly A.M., Chu K.H., Kinnear F.B., Keijzers G., Laribi S. (2022). Migraine in the Emergency Department: A Prospective Multinational Study of Patient Characteristics, Management, and Outcomes. Neuroepidemiology, 56(1), 32-40	ED study (HEAD study)

Indirect costs

Datasets/studies reviewed

- General Social Survey
- Household Disability Survey
- Household Economic Survey
- Household Labour Force Survey
- NZ Income Survey
- Household Income and Living Survey
- Quarterly Employment Survey
- Linked Employee-Employer Database
- New Zealand Census of Population and Dwellings
- International Social Survey Programme (ISSP)
- New Zealand Health, Work and Retirement Study
- Te Kupenga: Post censal survey of Māori well being
- Time Use Surveys
- New Zealand Longitudinal Study of Ageing
- Te Puāwaitanga O Ngā Tapuwae Kia Ora Tonu/Life and Living in Advanced Age Cohort Study
- Living in Aotearoa
- Youth2000 Survey Series
- Youth, Health & Wellbeing Survey
- Survey of Family, Income and Employment (SoFIE)
- NZ Health Survey
- Migraine in Aotearoa NZ Survey
- Dunedin Multidisciplinary Health and Development Study
- Quality of Life Survey 2022 (NielsenIQ)

Studies screened from literature searches:

Article	Data source
Blakely T., Sigglekow F., Irfan M., Mizdrak A., Dieleman J., Bablani L., Wilson N. (2021). Disease-related income and economic productivity loss in New Zealand: A longitudinal analysis of linked individual-level data. PLoS Medicine, 18(11)	Administrative data
Chan J.K. & Consedine N.S. (2014). Negative affectivity, emotion regulation, and coping in migraine and probable migraine: a New Zealand case-control study. International Journal of Behavioral Medicine, 21(5), 851-860	Case control study – no indirect cost information
Chu K., Kelly A.-M., Kinnear F., Keijzers G. & Kamona S. (2022). Primary headache drug treatment in emergency departments in Australia and New Zealand. Medical Journal of Australia, 217(7), 366-367	ED study (HEAD study) – no indirect cost information
Garrett, Susan, and Fiona Imlach. (2024). The Impact of Living with Migraine Disease in Aotearoa New Zealand. New Zealand Medical Journal 137 (1592): 54–76	Patient survey (Migraine in Aotearoa NZ)
Imlach F. & Garrett S. (2025). Use of Non-Pharmacological Approaches for Migraine Treatment: Results from the Migraine in Aotearoa New Zealand Survey. Journal of Clinical Medicine, 14(12)	Patient survey (Migraine in Aotearoa NZ) – no indirect cost information
Kelly A.M., Kuan W.S., Keijzers G., Karamercan M.A., Wijeratne T., Graham C.A., Russell K. (2021). Epidemiology, investigation, management, and outcome of headache in emergency departments (HEAD study)-A multinational observational study. Headache, 61(10), 1539-1552	ED study (HEAD study) – no indirect cost information
McInnarney B., Imlach F., Kennedy J. & Garrett S.M. (2024). Patient perceptions of barriers to effective migraine management in Aotearoa New Zealand. Journal of Primary Health Care, 16(4), 347-356	Patient survey (Migraine in Aotearoa NZ) – no indirect cost information
Randerson J., Imlach F., Kennedy J. & Garrett S. (2025). Primary care clinicians' perspectives on migraine management in Aotearoa New Zealand: a qualitative study. Journal of Primary Health Care	Qualitative study of primary care clinicians – no indirect cost information
Summers, Jennifer A., Nick Wilson, Tony Blakely, and Finn Sigglekow. 2023. Disease-Related Loss to Government Funding: Longitudinal Analysis of Individual-Level Health and Tax Data for an Entire Country. Value in Health 26 (2): 170–75	Administrative data
Waldie KE & Poulton R (2002). The burden of illness associated with headache disorders among young adults in a representative cohort study. Headache, 42(7), 612-9	Dunedin Study (birth cohort)

Supplementary Material C: Evaluation of migraine prevalence studies

Study (date of data collection)	Sampled population	Sampling method	Number of respondents	Participation rate	Access	Validation of diagnostic instrument	Diagnostic criteria	Prevalence timeframe	Total score[1]	Māori and Pacific representation	Availability of dataset and linkage
Thomson (1990)	3	3	1	3 (79%)	3	-4	2	4	15	Yes	Not available
Dunedin study (1998/99)	3	3	1	4 (96%)	3	-4	2	4	16	No	Not available
Auckland Birthweight study (2008/10)	3	3	1	3 (71% of those enrolled at birth)	3	-4	2	4	15	No	Not available
SoFIE (2004/05)	4	4	4	2 (77% in wave 1, 61% in wave 3)[36]	3	-4	1	4	18	Yes	Available on application through IDI
NZHS (2006/07)	4	4	4	2 (68%)	3	-4	1	4	18	Yes	Available on application through IDI
NZHS (2013/14)	4	4	4	4 (80%)	3	-4	1	4	18	Yes	Available on application through IDI
NZHS (2023/24)	4	4	4	3 (73%)	3	-4/3[2]	1	4	18/27[2]	Yes	Available on application through IDI

GBD (2023)									No	Not available
GBD (2021)									Māori	Not available

[1] Possible range is −27 to +32. Quality thresholds: <9: unacceptable (data of no value); 9–23: acceptable when no other data are available; 24–27: good; ≥28: very good

[2] Two different assessments of migraine in this survey; one using a tool validated in clinic populations (ID-Migraine)

Quality scoring criteria

From: Stovner LJ, Al Jumah M, Birbeck GL, et al. The methodology of population surveys of headache prevalence, burden and cost: principles and recommendations from the Global Campaign against Headache. *J Headache Pain*. 2014;15(100940562):5. doi:10.1186/1129-2377-15-5

**Quality factor**	**Score**				
	**−4**	**1**	**2**	**3**	**4**
**Sampled population**	Not stated, or clinic population, or members of patient organization	Selected population (eg, health-plan members, work-place, college students) [increase score by 2 if this met the specific purpose of the study]		General population or community-based sample from defined region within a country, or school-based (for children)	General population or community-based sample from whole country
**Sampling method**	Not stated	No (or failed) attempt to secure representativeness		Random sample uncorrected for population demographics	Total defined population, or random sample corrected for population demographics
**Number of respondents**	Not stated	250-1,000	1,000-1,500	1,500-2,500	>2,500
**Participation rate**	Not stated, or <40%	50-59%	60-69%	70-79%	>80%

**Access**	Not stated	Self-administered (unsupervised) questionnaire	Telephone or face-to-face interview by untrained or unspecified interviewer(s)	Telephone or face-to-face interview by trained lay interviewer(s), medical students or nurses	Face-to-face interview with headache expert [reduce score by 1 if interviews restricted to screen-positive sub-sample]
**Validation of diagnostic instrument**	Instrument not specified or not validated	Validated, but sensitivity and/or specificity <60%	Validated, but sensitivity and/or specificity <70%	Validated only in screen-positive sub-sample, or in clinic or unspecified sample, but sensitivity and specificity ≥70%	Validated in target population or similar, and sensitivity and specificity ≥70%, or all diagnoses made in face-to-face or telephone interviews by headache expert
**Diagnostic criteria, and application of "probable" diagnoses**	Not stated	Stated, other than ICHD	ICHD (or reasonable modification), but uncertain or inappropriate analysis of "probable" diagnoses		ICHD (or reasonable modification) with clear exposition regarding "probable" diagnoses
**Prevalence time frame**		Not specified or not appropriate to the study purpose	Not specified, but terminology implies "present" or "current" or "recent" headache	Other specified period appropriate to the study purpose	Point, 1-day, 1-year or lifetime
